# Microbiologically influenced corrosion of stainless steel independent of sulfate-reducing bacteria

**DOI:** 10.3389/fmicb.2022.982047

**Published:** 2022-10-12

**Authors:** Satoshi Wakai, Nanami Eno, Hirotaka Mizukami, Toshiyuki Sunaba, Kazuhiko Miyanaga, Yasuyuki Miyano

**Affiliations:** ^1^Institute for Extra-Cutting-Edge Science and Technology Avant-Garde Research (X-Star), Japan Agency for Marine-Earth Science and Technology (JAMSTEC), Yokosuka, Japan; ^2^PRESTO, Japan Science and Technology Agency (JST), Tokyo, Japan; ^3^Materials and Corrosion Group, Technical Research Center, Technical Division, INPEX Corporation, Tokyo, Japan; ^4^School of Life Science and Technology, Tokyo Institute of Technology, Yokohama, Japan; ^5^Graduate School of Engineering Science, Akita University, Akita, Japan

**Keywords:** microbiologically influenced corrosion, biofilm, microbial community, stainless steel, *Nitrospira*, *Beggiatoaceae*

## Abstract

The presence and activities of microorganisms on metal surfaces can affect corrosion. Microbial communities after such corrosion incidents have been frequently analyzed, but little is known about the dynamics of microbial communities in biofilms on different types of stainless steels, such as austenitic, martensitic, and duplex stainless steels. Here, we conducted immersion experiments on 10 types of stainless steels in a freshwater environment, where microbiologically influenced corrosion was observed. During 22-month of immersion, severe localized corrosions were observed only on martensitic S40300 stainless steel. Microbial community analysis showed notable differences between non-corroded and corroded stainless steels. On the surfaces of non-corroded stainless steels, microbial communities were slowly altered and diversity decreased over time; in particular, relative abundance of *Nitrospira* sp. notably increased. Whereas microbial communities in corrosion products on corroded stainless steels showed low diversity; in particular, the family *Beggiatoaceae* bacteria, iron-oxidizing bacteria, and *Candidatus* Tenderia sp. were enriched. Furthermore, sulfur enrichment during localized corrosion was observed. Since there was no enrichment of sulfate-reducing bacteria, the sulfur enrichment may be derived from the presence of family *Beggiatoaceae* bacteria with intracellular sulfur inclusion. Our results demonstrated slow and drastic changes in microbial communities on the healthy and corroded metal surfaces, respectively, and microbial communities on the healthy metal surfaces were not affected by the composition of the stainless steel.

## Introduction

Stainless steel exhibits resistance to corrosion in moderate environments, such as freshwater, neutral pH, and ambient atmosphere and temperature. This is because of the formation of a passive film on the surface. However, this passive film is degraded by halide ions such as chloride and bromide (Malik et al., 1992; Dastgerdi et al., 2019); thus, general-purpose stainless steels corrode in salty environments. The UNS S30400 and S31600 stainless steels have been used in various environments as general-purpose stainless steels. Both stainless steels are austenitic stainless steels with different chemical compositions; S31600 is more corrosion-tolerant than S30400 because of its higher Cr and Ni content and the addition of Mo (Table 1). Similarly, various austenitic stainless steels exist and their metal compositions differ. Different compositions have different mechanical properties and corrosion resistances (Baddoo, 2008). In addition to austenitic stainless steels, martensitic and duplex stainless steels have different metal phases; martensitic S40300 and duplex S31260 stainless steels are less and more corrosion-tolerant, respectively, than austenitic stainless steels. Therefore, various types of stainless steels have been used in suitable environments based on their mechanical and corrosion-resistance properties.

**Table 1:** Metals used in the immersion study.

	Grade	Engraving	C	Si	Mn	Ni	Cr	Mo	Cu	W	Fe
Austenitic	S30400	S8	≤0.08	≤1.00	≤2.00	9.25	19.00	–	–	–	Bal.
	S31600	S6	≤0.08	≤1.00	≤2.00	12.00	17.00	2.50	–	–	Bal.
	Casting S31600	S1	≤0.08	≤1.50	≤1.50	10.50	19.50	2.50	–	–	Bal.
	S30300	S3	≤0.15	≤1.00	≤2.00	9.0	18.0	≤0.60	–	–	Bal.
Martensitic	S40300	S4	≤0.15	≤0.50	≤1.00	–	12.25	–	–	–	Bal.
	S42000	L8	0.2	0.5	0.44	0.1	13.0	–	–	–	Bal.
	S41426	HP	0.03	0.3	0.46	5	13.1	2	–	–	Bal.
	15% Cr	5C	0.03	0.2	0.28	6.3	14.7	2.0	1.0	–	Bal.
Duplex	17% Cr (Martenstic-ferritic)	7C	0.03	0.2	0.37	3.9	16.5	2.4	1.0	1.0	Bal.
	S312600 (austenitic/ferrittic)	J	≤0.03	≤1.00	≤1.50	6.50	25.00	3.00	–	–	Bal.

Despite their suitable grade and proper usage, corrosion of stainless steels has sometimes been observed under moderate conditions, and it is attributed to microbiologically influenced corrosion (MIC; Ibars et al., 1992; Iversen, 1999; Linhardt and Nichtawitz, 2003). Reports on the corrosion of stainless steel are relatively fewer than those of carbon steel. Furthermore, inclusion of manganese sulfide is reported to chemically influence pitting corrosion (Ryan et al., 2002); however, little is known about corrosive microorganisms in the MIC of stainless steel. There are some studies on the MIC by sulfate-reducing bacteria (SRB; Ringas and Robinson, 1988; Webster and Newman, 1993; Antony et al., 2007; Xu et al., 2007; Li et al., 2009; Sun et al., 2011; Yuan et al., 2013; Liang et al., 2014; Zhang et al., 2015; Wu et al., 2019, 2021). Moreover, we recently reported dynamic changes in microbial communities during the corrosion of some steels and the absence of SRB in the early corrosion phase (Wakai et al., 2022), indicating that the detection and enrichment of SRB alone does not represent the corrosion process in the MIC.

There are recent reports of various novel iron-corrosive microorganisms (Dinh et al., 2004; Mori et al., 2010; Uchiyama et al., 2010; McBeth et al., 2011; Wakai et al., 2014; Iino et al., 2015; Kato et al., 2015; Hirano et al., 2022) and the corrosion mechanisms of some corrosive microorganisms were also reported (Deng et al., 2015; Tsurumaru et al., 2018). Most of those microorganisms are capable of corroding zero-valent iron and carbon steel but not stainless steels under laboratory-scale conditions. MIC is divided into chemical MIC (CMIC) and electrical MIC (EMIC; Enning and Garrelfs, 2014). CMIC is indirect corrosion by microbially produced substances, and the rate-limiting reaction in CMIC under anaerobic conditions is H_2_ production by the reduction of protons (H^+^; Eq. 1); namely, the corrosion rate based on the oxidation of Fe^0^ (Eq. 2) is nearly equal to the H_2_ production rate.


(1) $${\mathrm{2H}}^{+}+2\;e^{-}=H_{2}$$

(2) $${\mathrm{Fe}}^{0}={\mathrm{Fe}}^{2+}+2\;e^{-}$$

Whereas, EMIC is direct corrosion caused by the consumption of electrons from metals and is promoted by extracellular electron transfer (EET; Liu et al., 2018). EET-promoted microbial corrosion is faster than other microbial corrosions and proceeds by EET metabolism independent of H_2_ production, which is the rate-limiting reaction in CMIC. For examples, rates of sulfate reduction (Eq. 3) without the H_2_ production would correspond to the corrosion rate.


(3) $${\mathrm{SO}}_{4}{}^{2-}+8\;H^{+}+8\;e^{-}=S^{2-}+4\;H_{2}O$$

Laboratory-scale corrosion experiments using electrochemically active microorganisms have been reported recently (Miller et al., 2018; Dou et al., 2019; Kalnaowakul et al., 2020; Tang et al., 2021; Huang et al., 2022). Because various microorganisms live in complex community structure in the actual environments, understanding the mechanism of MIC by a single microorganism is inadequate. Further, little is known about the microbial community dynamics during the corrosion of stainless steels and the formation of biofilms. Therefore, we focused on the corrosion behavior and biofilm formation using various types of stainless steels in the same environment.

Therefore, we studied the MIC by immersing 10 types of stainless steels in an aerobic freshwater environment with a history of MIC incidents. Test pieces were withdrawn at definite intervals, and the corrosion rate of each metal and the structure of microbial community were investigated. Our findings provide insights into the maturation of microbial communities in biofilms on stainless steel and the MIC independent of SRB.

## Materials and methods

### Immersion experiment

Ten types of stainless steels were used in this experiment (Table 1). They were machined into 50 mm × 20 mm × 1–5 mm blocks (thicknesses of S42000, S41426, 15% Cr, and 17% Cr stainless steels: 5 mm; thicknesses of S30300 stainless steel: 4 mm; and thicknesses of Casting S31600, S40300, and S312600 stainless steels: 3 mm) with two 4 mm holes and polished with 600 grit emery paper. All coupons were washed ultrasonically with 99.5% ethanol and acetone, dried, and finally weighted. Polytetrafluoroethylene rods were inserted into the stainless-steel coupons separated by spacers (Diameter × height: 5 mm × 30 mm). The assembly resembled a ladder, and 10 sets of such ladders were prepared: five sets of ladders were used to determine the corrosion rate, and the remaining ladders were used for microbiome analysis.

The immersion experiment was performed in an indoor industrial water storage pool (approximately 1,100 m^3^) with 20 m^3^/h inflow and overflow-type drainage. Industrial water is supplied by treating river water, and the quality was as follows: 10–30 ppm Cl^−^, 10–30 mS/m, 20–50 ppm Ca^2+^, 10–30 ppm SiO_2_, 1 ppm turbidity, pH 7.4, and ~ 8.2 ppm dissolved oxygen. As the pool did not have any temperature control system, the temperature varied ranging from 9 to 23°C.This arrangement is the same as that reported in Wakai et al. (2022). Two sets of ladders were withdrawn from the pool every 1, 3, 6, 14, and 22 months; the pieces from one of the ladders were used to determine the weight and corrosion rates and those from the remaining ladder were used for microbiome analysis.

### Calculation of corrosion rates

The corrosion products and biofilms on the test pieces were removed by scraping with a plastic spatula or wiping with a cotton swab, respectively, and were washed in 99.5% ethanol using an ultrasonic bath. The samples were then immersed in Clarke’s solution according to ASTM standard G1-03 (ASTM G1-03, 2003). All test pieces were weighed after complete drying. The corrosion rate of each test piece was calculated using the following equation:


(4) Corrosion rate=(K×W)/(A×T×D)

where corrosion rate is expressed in mm/y, K is a constant (8.76 × 10^4^), T is the time of exposure in h, A is the area in cm^2^, W is the weight lost in g, and D is the density in g/cm^3^.

After weighing the samples, 3D images of some coupons were captured using a 3D measuring laser microscope (LEXT OLS4000, Olympus Corp., Tokyo, Japan). Further, the corroded coupons were analyzed by electron probe microanalysis (EPMA) before washing with Clarke’s solution. Healthy surfaces, bare metal surfaces under corrosion products, and corrosion pit inclusions were analyzed using an EPMA (JXA-8230; JEOL, Tokyo, Japan) with an acceleration voltage of 15.0 kV and irradiation current of 50 nA in qualitative analysis mode.

### Microbial community analysis

#### DNA extraction

Each sample withdrawn from the pool was transferred to a polyethylene bag and immediately frozen using dry ice pellets. DNA from the biofilm and corrosion products on the metal surface were extracted following the methods of Wakai et al. (2022). 1.8 ml of DNA/RNA shield solution (Zymo Research, Irvine, CA, United States) was poured into the bag, and the corrosion products and attached cells were recovered by washing the bag. The solution was centrifuged at 10000 × *g* for 5 min, and the supernatant was discarded. The pellet consisting of corrosion products and microbial cells was resuspended in fresh DNA/RNA shield solution, and the DNA was extracted using the ZymoBIOMICS™ DNA/RNA Miniprep Kit (Zymo Research) following the manufacturer’s protocol. DNA concentrations were measured using a Qubit™ dsDNA HS assay kit (Thermo Fisher Scientific, Waltham, MA, United States) and a Qubit 4 Fluorometer (Thermo Fisher Scientific).

#### Amplicon sequencing of 16S rRNA gene fragment

Partial 16S rRNA genes (V4–V5 regions) were amplified by PCR using the primer sets 530F and 907R (Caporaso et al., 2012), which contain overhung adapters at the 5′ ends. PCR amplification, enzymatic purification, addition of multiplexing indices and Illumina sequencing adapters, and purification with magnetic beads were performed as previously described (Hirai et al., 2017). Amplicon sequencing was performed using an Illumina MiSeq platform and a MiSeq v3 reagent (Illumina, United States) as the 300 bp paired end following Illumina’s standard protocol.

Raw FASTQ files generated by MiSeq were analyzed using the QIIME2 pipeline (Bolyen et al., 2019). Pair-end raw FASTQ files were demultiplexed using the demux plugin, based on their unique barcodes (Caporaso et al., 2010). Demultiplexed sequences from each sample were processed using the dada2 plugin to obtain a feature table (Callahan et al., 2016). The feature-classifier plugin was then used to align feature sequences to a pre-trained SILVA-138 99% database to generate a taxonomy table (Bokulich et al., 2018). The data were rarefied prior to alpha and beta diversity analyses, at a depth of 40,565 reads. Diversity metrics were calculated and plotted using the core-diversity plugin and Emperor plugin (Vázquez-Baeza et al., 2013). To statistically show the variability of the data, the box-and-whisker plots was prepared using the Excel plugin.

## Results

### Corrosion behavior

**Figure 1:**
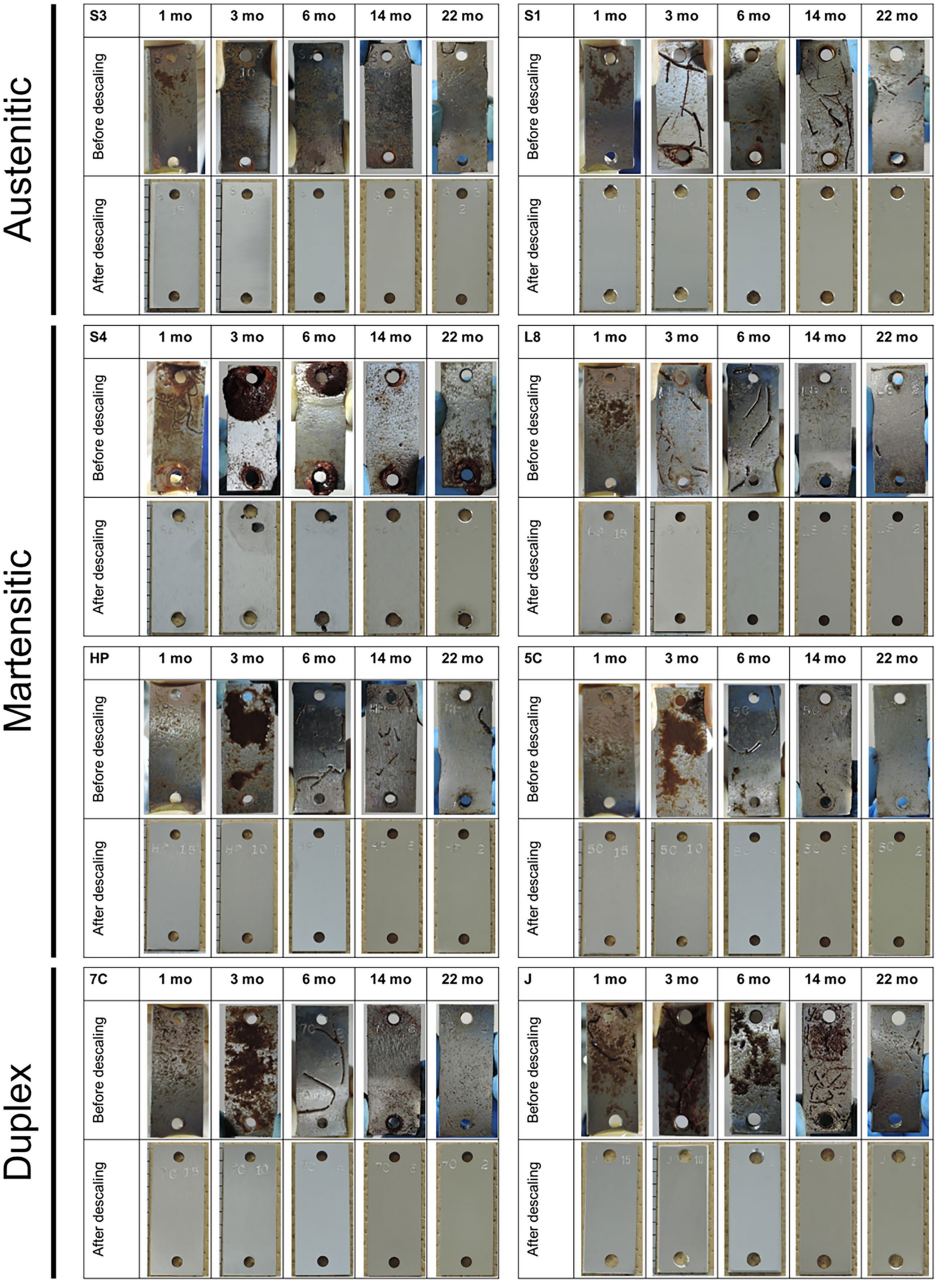
Coupons after immersion study. Macroscopic images of each coupon (50 mm high × 20 mm wide) are shown before and after descaling. S1, Casting S31600; S3, S30300; S4, S40300; L8, S42000; HP, S41426; 5C, 15% Cr; 7C, 17% Cr; and J, S31260.

Brown deposits were observed on the surface of all coupons recovered at 1 and 3 months, which were not corrosion products; the surface under the deposits was healthy. However, pitting as localized corrosion was observed near the 4-mm-holes in S40300 stainless steel (Figure 1).

**Figure 2:**
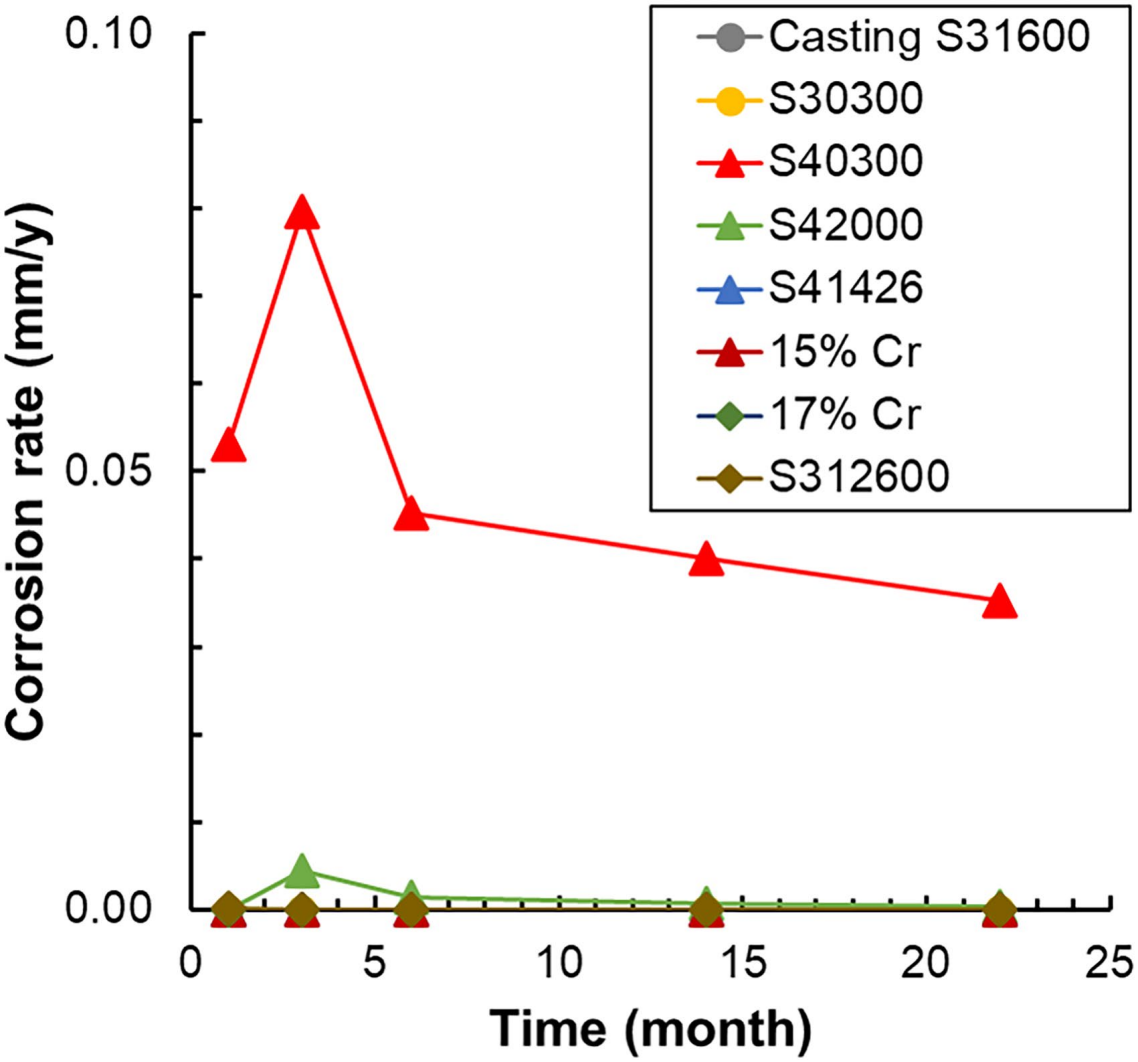
Variation in the corrosion rate of each metal with time. The corrosion rates are calculated using weight loss and immersion time. S1, Casting S31600; S3, S30300; S4, S40300; L8, S42000; HP, S41426; 5C, 15% Cr; 7C, 17% Cr; and J, S31260.

**Figure 3:**
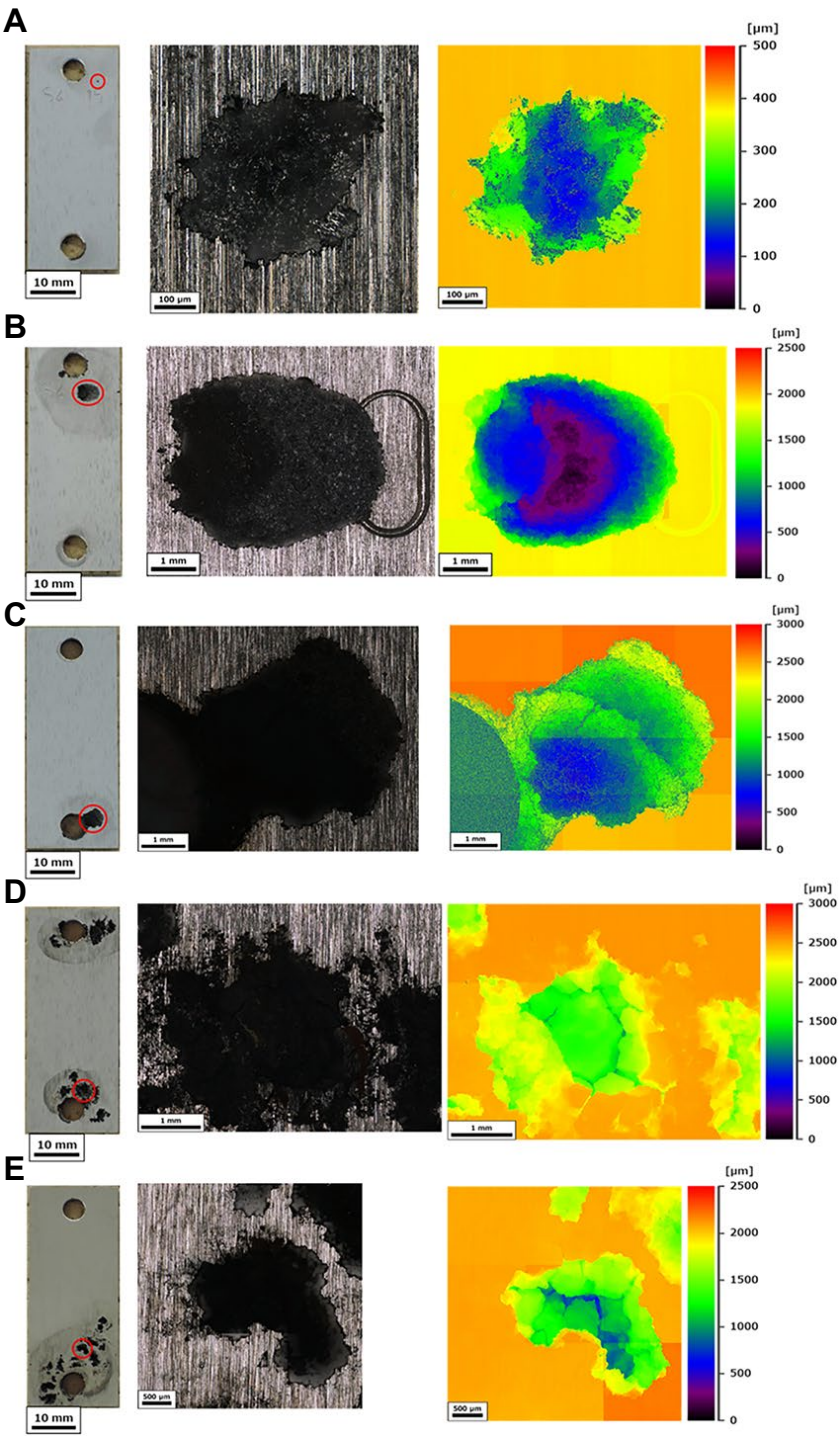
Corrosion of S40300 stainless steel. The full view (scale bars: 10 mm) and localized corrosions with maximal depths were visualized using 3D measuring laser microscopy at 1 month **(A)** 3 months **(B)** 6 months **(C)** 14 months **(D)** and 22 months **(E)**. The red circles in the full views indicate the measured localized corrosions; mo: month(s).

**Figure 4:**
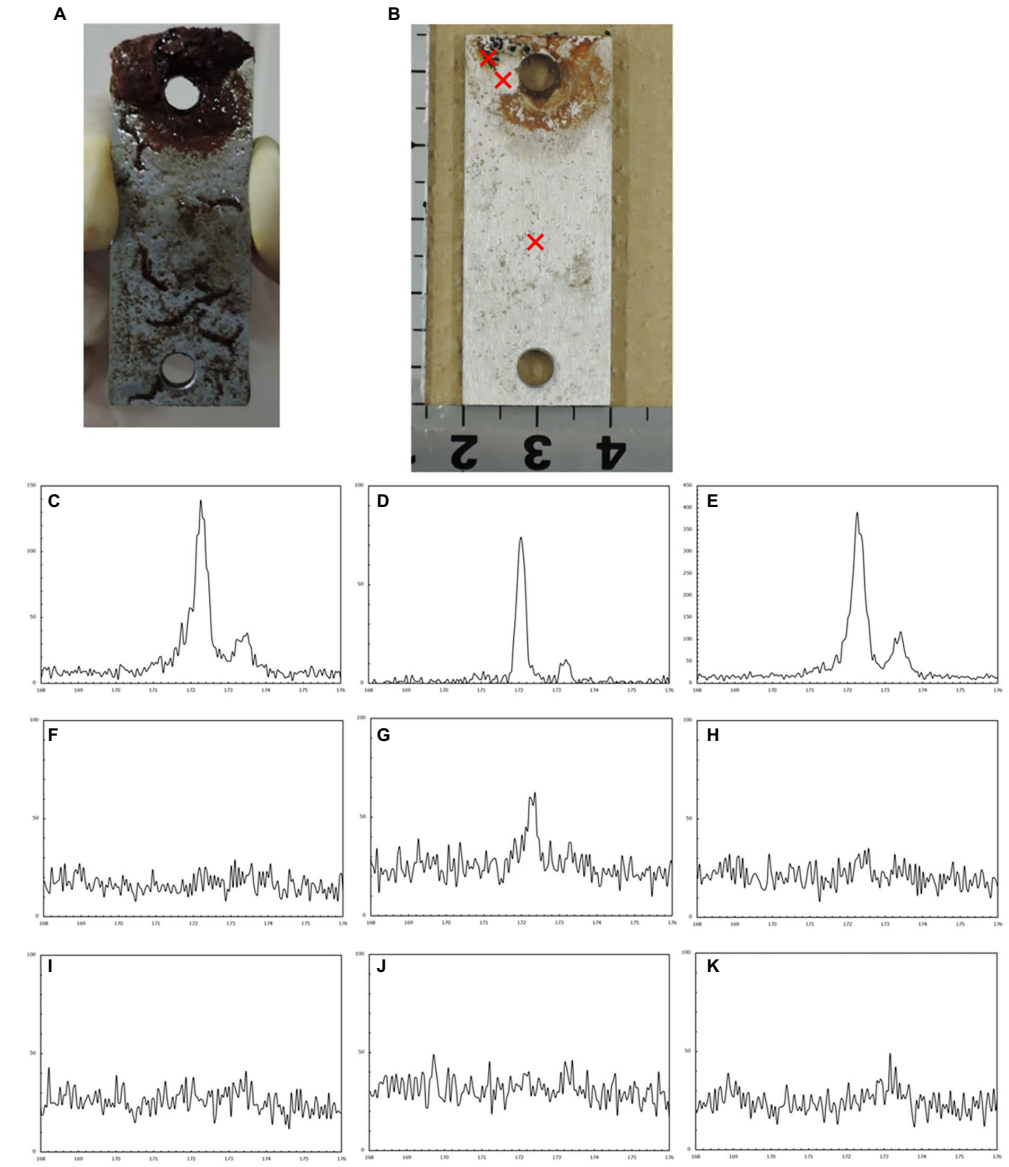
Electron probe microanalysis (EPMA) analysis of the corroded S40300 stainless steel coupon. Macroscopic images before **(A)** and after **(B)** washing. The cross symbols represent the sites of spot analysis. EPMA spectra of each spot: corrosion pit inclusion **(C–E)** bare surface under the corrosion products **(F–H)** and healthy surface without the corrosion products **(I–K)**.

The corrosion rates determined from the weight loss in the S40300 stainless steels were 0.04–0.08 mm/y (Figure 2). Corrosion products were developed on the coupons at 3 and 6 months (Figure 1), and deep pitting corrosion was observed under the corrosion products (Figure 3). The maximum corrosion depths at 1, 3, 6, 14, and 22 months were 305, 1,892, 1904, 1,247, and 1,477 μm, respectively, and the localized corrosion rates estimated using the corrosion depth and immersion time at 1, 3, 6, 14, and 22 months were 3.84, 8.12, 3.84, 1.10, and 0.80 mm/y, respectively. At 3 and 6 months, the number and diameter of pits were smaller and larger (~5 mm), respectively, compared with those at 14 and 22 months. Furthermore, the inside of the pitting, bare metal under the corrosion products, and healthy surface were analyzed by EPMA (Figures 4A,B). A peak of sulfur in the pitting corrosion was detected (Figures 4C–E) but not on the healthy surfaces (Figures 4F–K). This result clearly showed the enrichment of sulfur during the pitting corrosion.

### Alpha diversity of microbial communities

Amplicon sequencing was conducted to understand the differences in microbial communities in biofilms on metal surfaces over time. In 40 samples, 894,482 reads from S30400 and S31600 stainless steels (accession numbers: DRR334907–DRR334916; Wakai et al., 2022) and 2,774,371 reads, ranging from 40,565 to 128,474, from the rest were obtained. Thus, from a total of 3,668,853 reads, alpha and beta diversity and taxonomy plots were analyzed.

**Figure 5:**
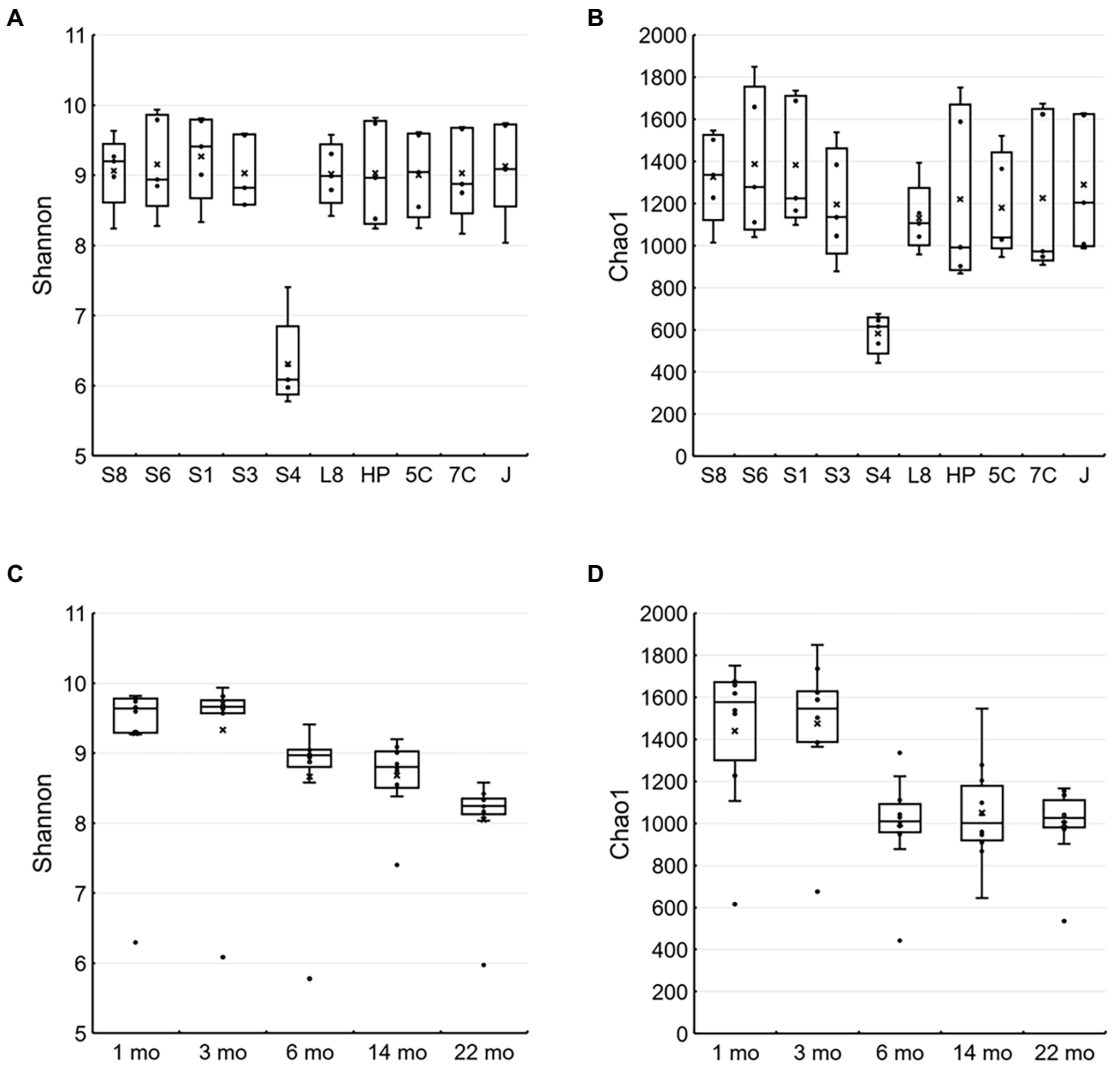
Alpha diversity of microbial communities in each environment and metal. The Shannon **(A,C)** and Chao1 **(B,D)** indices are represented as box-and-whisker plots. **(A)** and **(C)** were calculated based on each type of stainless steel (*n* = 5), and **(B)** and **(D)** were calculated based on time of each sampling (*n* = 10). The line in the middle of the box, top, and bottom of the box, whiskers, and cross symbols represent the median, 25 and 75 percentiles, min-to-max values, and average, respectively. S8, S30400; S6, S31600; S1, Casting S31600; S3, S30300; S4, S40300; L8, S42000; HP, S41426; 5C, 15% Cr; 7C, 17% Cr; and J, S31260.

Shannon and Chao1 indices as alpha diversity of biofilms on all samples except for the S40300 stainless steel showed 8.03–9.93 and 868–1850, respectively, and those for the S40300 stainless steel were 5.78–7.40 and 442–674, respectively (Figures 5A,B). This suggests that the microbial diversity in the biofilm on S40300 stainless steel was lower than that on the other metals. Furthermore, the Shannon indices of the biofilm on the metals, except for S40300 stainless steel, decreased from approximately 10–8 over time (Figure 5C). This suggests that the microbial diversity of biofilms on healthy stainless-steel surfaces gradually decreased.

### Beta diversity of microbial communities

**Figure 6:**
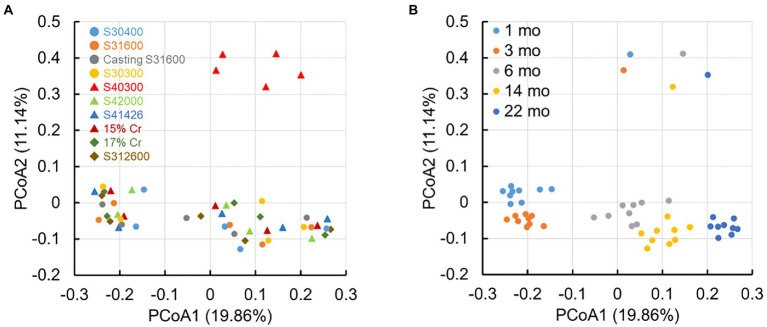
Principal coordinate analysis (PCoA) plots based on the unweighted UniFrac distances. S8, S30400; S6, S31600; S1, Casting S31600; S3, S30300; S4, S40300; L8, S42000; HP, S41426; 5C, 15% Cr; 7C, 17% Cr; and J, S31260.

Figure 6 shows the principal coordinate analysis (PCoA) plot based on the unweighted UniFrac distances of all samples. The plots of microbial communities from S40300 stainless steel were distinctly separate from the other plots (Figure 6A). In addition, the plots of those from other stainless steels are divided into two clusters: one consists of the plots from 1 and 3 months, and the other consists of plots from 6, 14, and 22 months (Figure 6A). In the cluster consisting of plots at 6, 14, and 22 months, these plots shifted chronologically to one direction (Figure 6B). This suggests that the microbial communities on the S40300 stainless steel clearly differed from those in biofilms on the healthy stainless steels, and the microbial communities on the healthy stainless steels changed over time.

### Microbial communities at the phylum level

**Figure 7:**
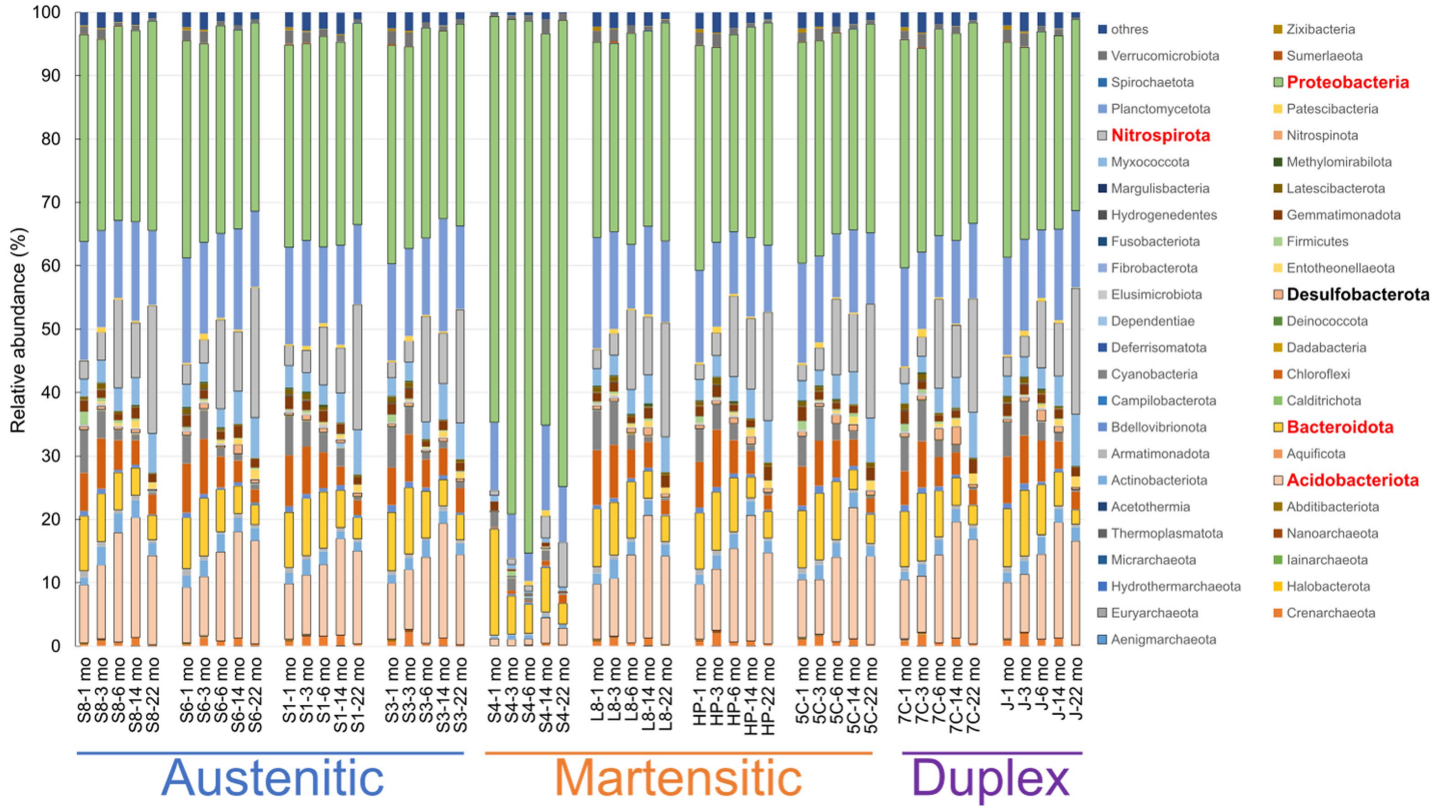
Relative abundances of microorganisms at phylum level in each sample. S8, S30400; S6, S31600; S1, Casting S31600; S3, S30300; S4, S40300; L8, S42000; HP, S41426; 5C, 15% Cr; 7C, 17% Cr; and J, S31260.

The major phyla of microbial communities in all samples were *Proteoacteria* (29.6–83.9%), *Acidobacteriota* (1.0–20.7%), *Nitrospirota* (0.7–20.5%), *Planctomycetota* (4.4–18.7%), and *Bacteroidota* (2.2–16.7%; Figure 7). The relative abundances (61.6–83.9%) of *Proteobacteria* in S40300 stainless steel were notably higher than those (29.6–36.0%) of other stainless steels (Figure 7). In contrast, the relative abundances (1.0–4.0%) of *Acidobacteriota* in S40300 stainless steels were clearly lower than those (8.6–20.7%) in other stainless steels (Figure 7). Similarly, the relative abundances of *Nitrospirota*, *Chloroflexi*, *Myxococcota*, *Actinobacteriota*, and *Desulfobacterota* in S40300 stainless steel were lower than those in the other stainless steels (Figure 7).

**Figure 8:**
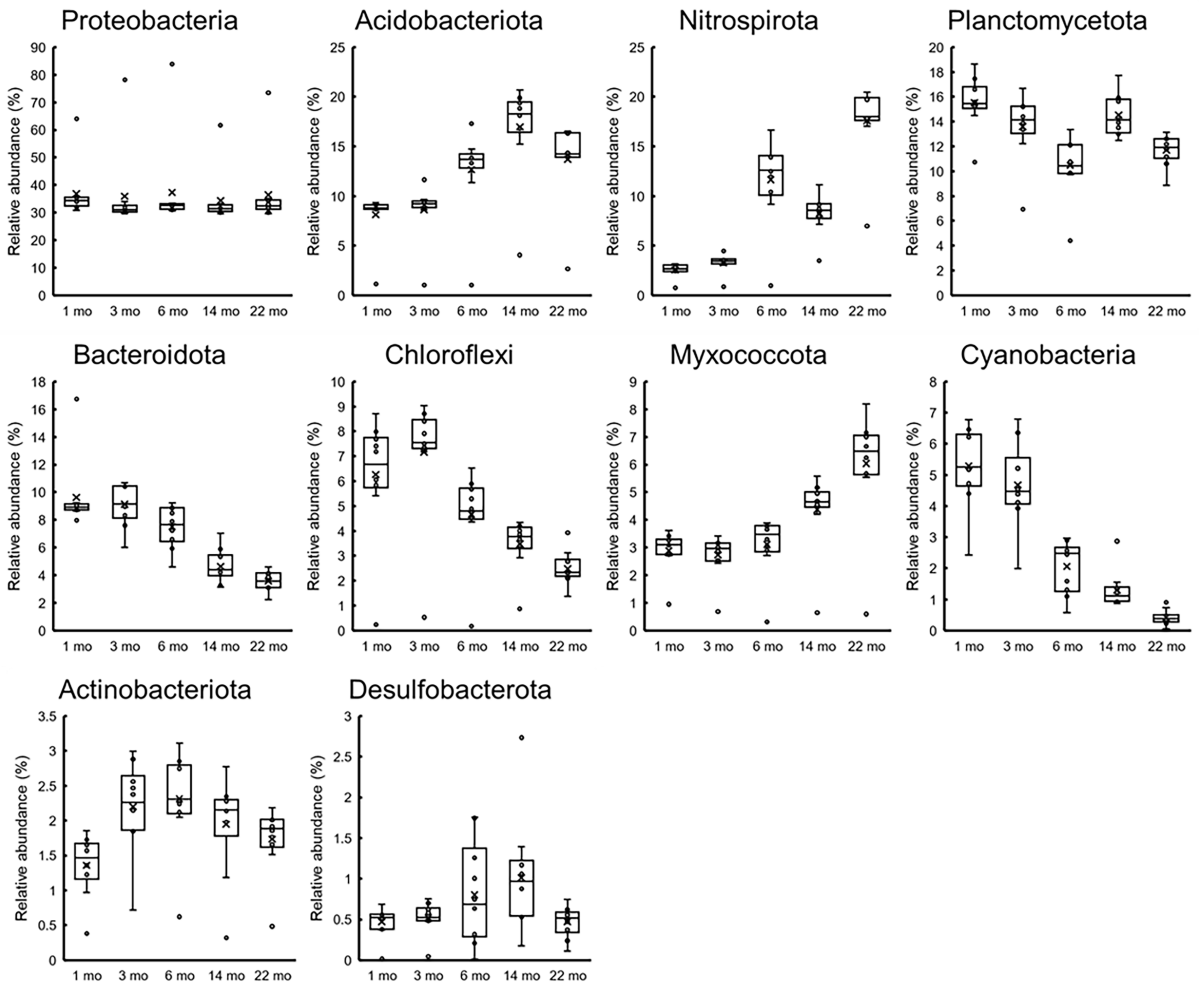
Temporal changes in 10 most abundant phyla. Relative abundances at the time of each sampling (*n* = 10) are represented as box-and-whisker plots. The line in the middle of the box, top and bottom of the box, whiskers, and cross symbols represent the median, 25 and 75 percentiles, min-to-max values, and average, respectively.

Figure 8 shows the changes in the relative abundances of 10 most abundant phyla over time. The abundances of *Acidobacteriota*, *Nitrospirota*, and *Mixococcota* increased, whereas those of *Planctomycetota*, *Bacteroidota*, *Chloroflexi*, and *Cyanobacteriia* decreased. The tendencies were more pronounced for healthy stainless steels, and almost all data from the corroded S40300 stainless steels were identified as outliers. These results are reasonable for the shift in the microbial communities over time in the PCoA plot (Figure 6).

### Transition of microbial communities at the genus level

**Figure 9:**
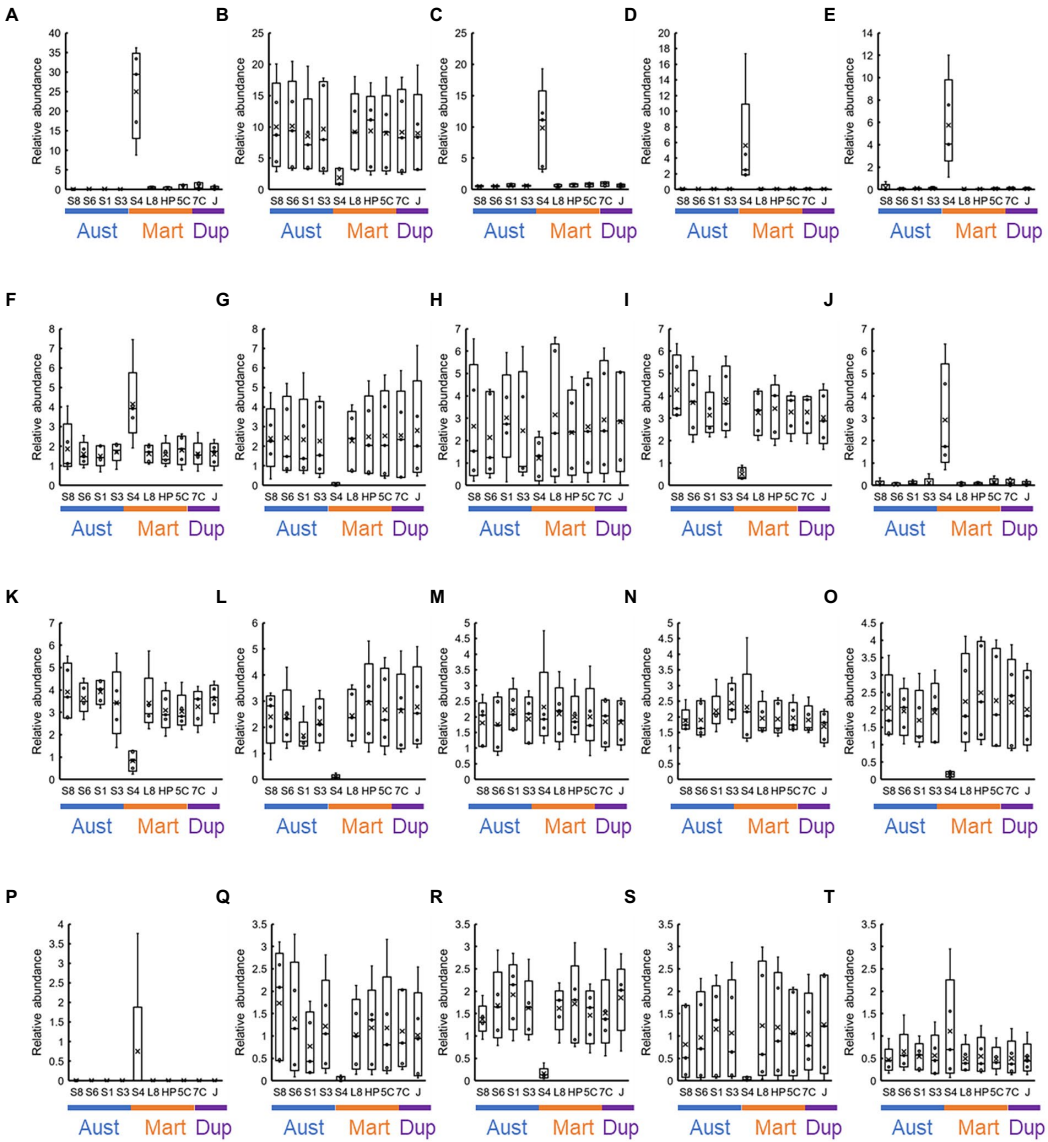
Co-abundance analysis of the 20 most abundant OTUs in each stainless steel. Relative abundances at the genus level in each stainless steel (*n* = 5) are represented as box-and-whisker plots. The line in the middle of the box, top and bottom of the box, whiskers, and cross symbols represent the median, 25 and 75 percentiles, min-to-max values, and average, respectively. **(A)** Family *Beggiatoaceae*; **(B)** *Nitrospira*; **(C)** class *Gammaproteobacteria*; **(D)** *Candidatus* Tenderia; **(E)** family *Sideroxydans*; **(F)** family *Comamonadaceae*; **(G)** phylum *Myxococcota*; **(H)** class *Cyanobacteriia*; **(I)** *Vicinamibacteraceae*; **(J)** *Ferritrophicum*; **(K)** family *Gemmataceae*; **(L)** order *Vicinamibacterales*; **(M)** family *Microscillaceae*; **(N)** phylum *Planctomycetota*; **(O)** order *Burkholderiales*; **(P)** *Leptospirillum*; **(Q)** *Luteitalea*; **(R)** family *Nitrosomonadaceae*; **(S)** family *Anaerolineaceae*; and **(T)** order *Rhizobiales*; S8, S30400; S6, S31600; S1, Casting S31600; S3, S30300; S4, S40300; L8, S42000; HP, S41426; 5C, 15% Cr; 7C, 17% Cr; and J, S31260.

At the genus level, approximately 1,000 OTUs containing unassigned bacteria and archaea were observed. Of these, we focused on 20 most abundant OTUs (Figure 9), and these covered 27.6–71.8% in each sample. Highest abundance was 36.1% of the family *Beggiatoaceae* bacteria in the S40300 stainless steel at 22 months (Figure 9). The high abundance of family *Beggiatoaceae* bacteria was a feature of the S40300 stainless steels, and the relative abundances in other stainless steels were a maximum of 1.8% (Figure 9). Similarly, a high abundance of bacteria of class *Gammaproteobacteria*, genera *Candidatus* Tenderia and *Sideroxydans*, family *Comamonadaceae*, and genera *Ferritrophicum* and *Leptospirillum* was observed in the S40300 stainless steels (Figure 9). However, bacteria of genus *Nitrospira*, phylum *Myxococcota*, families *Vicinamibacteraceae* and *Gemmataceae*, orders *Vicinamibacterales* and *Burkholderiales*, genus *Luteitalea*, and families *Nitrosomonadaceae* and *Anaerolineaceae* were abundant in healthy stainless steels, except for the S40300 stainless steels (Figure 9).

**Figure 10:**
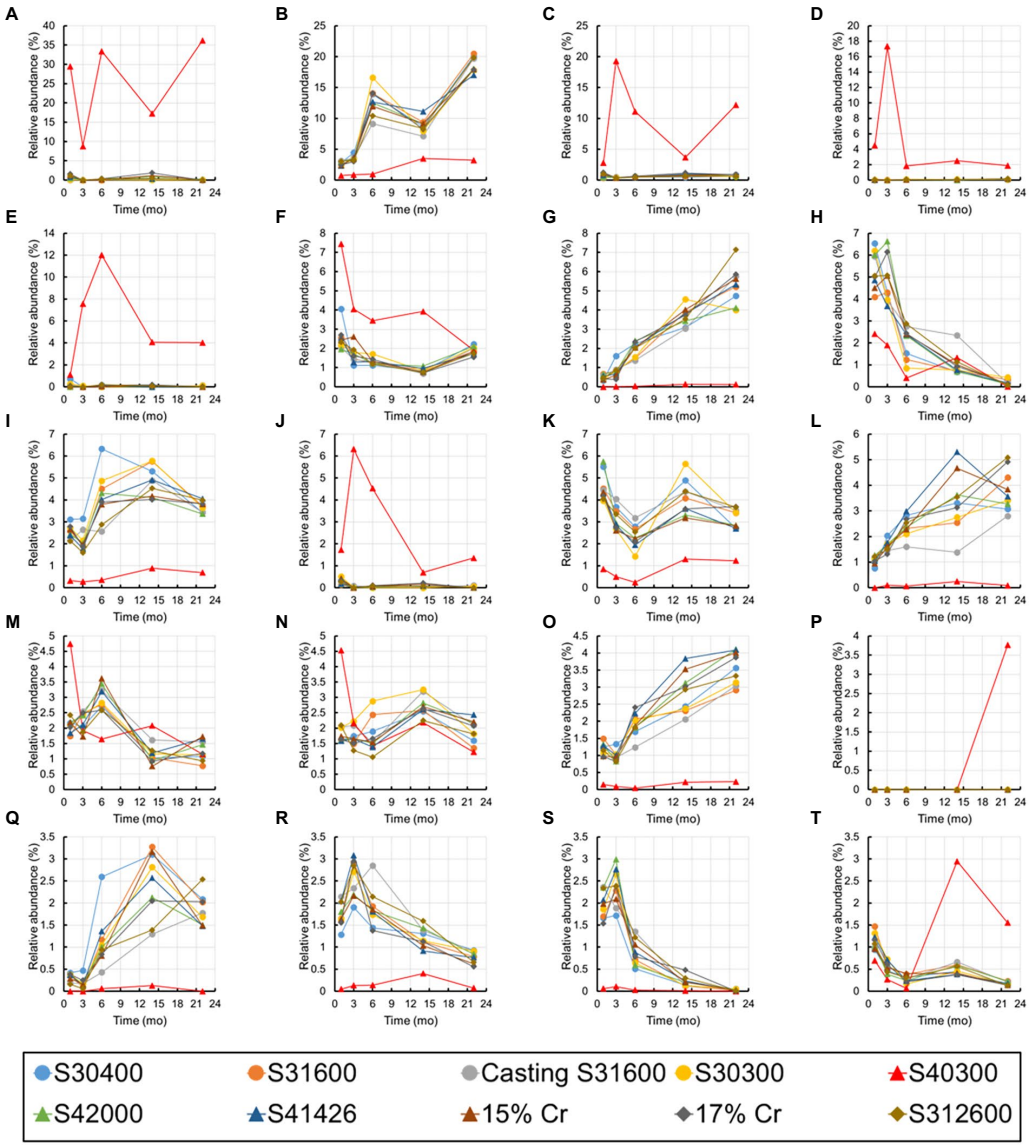
Temporal changes in the 20 most abundant OTUs. **(A)** Family *Beggiatoaceae*; **(B)** *Nitrospira*; **(C)** class *Gammaproteobacteria*; **(D)** *Candidatus* Tenderia; **(E)** family *Sideroxydans*; **(F)** family *Comamonadaceae*; **(G)** phylum *Myxococcota*; **(H)** class *Cyanobacteriia*; **(I)** family *Vicinamibacteraceae*; **(J)** *Ferritrophicum*; **(K)** family *Gemmataceae*; **(L)** order *Vicinamibacterales*; **(M)** family *Microscillaceae*; **(N)** phylum *Planctomycetota*; **(O)** order *Burkholderiales*; **(P)** *Leptospirillum*; **(Q)** *Luteitalea*; **(R)** family *Nitrosomonadaceae*; **(S)** family *Anaerolineaceae*; and **(T)** order *Rhizobiales*.

Figure 10 shows the changes over time in the 20 most abundant representations during the immersion experiment. As expected from Figure 9, the values from the S40300 stainless steels were plotted as outliers in almost all the cases. Levels of the bacteria in the genus *Nitrospira*, phylum *Myxococcota*, orders *Vicinamibacterales* and *Burkholderiales*, and genus *Luteitalea* increased, and those in class *Cyanobacteriia*, family *Nitrosomonadaceae*, and family *Anaerolineaceae* decreased over time (Figure 10). Of these, *Nitrospira* sp. increased by approximately 20%, and it was higher than the sum of relative abundances of the other four OTUs.

## Discussion

Many recent studies have reported biofilm formation and MIC using a single material and a single microbial species. In addition, several studies have reported the corrosion behavior by exposing various steel materials to the same corrosive environment. On the other hand, little is known about the transitions of microbial community structures on the biofilm and in the corrosion products. In this study, 10 types of stainless steels were immersed for 22 months in a freshwater environment and compared; the changes in the microbial community structure in their biofilms and corrosion behavior were investigated.

Ten types of stainless steels with different compositions and phases were used in this study. Of these, only S40300 stainless steel was corroded (Figures 1, 2); it is a martensitic stainless steel that does not contain Ni (Table 1). In contrast, the other martensitic stainless steels and austenitic and duplex stainless steels did not corrode. Higher Cr content and different phases could be the reason for the absence of corrosion in the austenitic and duplex stainless steels (Table 1). Higher Cr content in the S42000 steel and 15% Cr-stainless steel and Ni and Mo doping in the S41426 steel could have contributed to higher corrosion resistance compared with those of the S40300 stainless steel. Because our previous study showed corrosion in 9% Cr-steel immersed in freshwater (Wakai et al., 2022), the Cr-content in the S42000 steel in the present study may be close to the threshold value for corrosion susceptibility in this environment. Although S40300 stainless steel generally does not corrode in freshwater environments, localized corrosion in check valve plugs made from S40300 stainless steel has been observed in actual facilities. We suspect that this atypical corrosion of S40300 stainless steel in the freshwater environment could be because of MIC.

There are no reports on MIC and microbial community in the corrosion products and biofilms of S40300 stainless steel. Our data clearly showed the enrichment of *Beggiatoaceae* family bacteria in the corrosion products of S40300 stainless steel (Figure 9). We also observed the enrichment of *Beggiatoaceae* family bacteria in the corrosion products of 9% Cr steel but not in the corrosion products with low-Cr content and carbon steels in our previous study (Wakai et al., 2022). These data suggest that a high concentration of toxic chromium ions in the corrosion products influences the enrichment. In addition to the enrichment of the family *Beggiatoaceae*, accumulation of sulfur in the products of pitting corrosion was observed by EPMA (Figure 4). Although the accumulation of sulfur in the corrosion products has been frequently interpreted as the production of sulfide, SRB was hardly observed in the corrosion products of S40300 stainless steel (Figure 9). *Beggiatoa* sp. of the family *Beggiatoaceae* is known for sulfur-granule accumulation (Berg et al., 2014), and the accumulation of sulfur in the corrosion product may be because of the sulfur granules formed by family *Beggiatoaceae* bacteria. Such sulfur granules may influence corrosion by the CMIC mechanism because elemental sulfur is a reactive sulfur species (Schmitt, 1991).

In addition to the *Beggiatoaceae*, some bacterial genera, such as *Sideroxydans*, *Ferritrophicum*, and *Leptospirillum* were also enriched in the corrosion products of the S40300 stainless steel. As most of these microorganisms have iron-oxidizing properties, their enrichment is because of the energy acquisition by iron-oxidizing metabolism using Fe^2+^ generated during corrosion (Emerson et al., 2010). Furthermore, genus *Ca.* Tenderia was temporarily enriched in corrosion products, and it is an electroactive microorganism (Malanoski et al., 2018). Recent studies have reported EMIC by microorganisms with electrochemical activity (Enning and Garrelfs, 2014; Tang et al., 2021; Zhou et al., 2022); thus, we speculate that *Ca.* Tenderia may contribute to the cathodic reaction *via* electron consumption during the corrosion of S40300 stainless steel. Localized corrosion was particularly observed in the corrosion of S40300 stainless steel. The localized corrosion rates could reach 8 mm/y, suggesting a requirement of strong cathode reaction for such a higher corrosion rate.

Although stainless steels, except for S40300, did not corrode in this immersion experiment, we observed the maturation of microbial communities in the biofilm on the surfaces of healthy stainless steels. The enrichment of *Nitrospira* sp. was particularly a unique feature, and its relative abundance reached 20% (Figure 10). Such enrichment of *Nitrospira* sp. has been reported in microbial community analysis of biofilms and bulk water in a non-chlorinated drinking water distribution system model (Martiny et al., 2005). Presence of *Nitrospira* sp. has been detected in the *in situ* flow experiment using fresh dam water (Liao et al., 2010). *Nitrospira* sp. consumes nitrate and increases the open-circuit potential of stainless steel. Therefore, the presence and activity of *Nitrospira* sp. may correlate with the ennoblement of stainless steels, which would increase the risks of crevice and/or pitting corrosion.

In addition to *Nitrospira* sp., some organotrophic bacteria, such as the *Luteitalea* sp. (Vieira et al., 2017) and phylum *Myxococcota* (Waite et al., 2020) were enriched in the biofilm on the non-corroded stainless steels over time (Figure 10). Although the organic matter in the industrial water used in the present study was in trace quantities, the rate of water flow to the immersion pool was 20 m^3^/h. Such a continuous supply of trace quantities of organic matter could support a slow increase of organotrophic bacteria in the biofilms on the metal surfaces. In contrast, some bacteria, such as the families *Nitrosomonadaceae* and *Anaerolineaceae*, clearly decreased. The reason for this is unknown; further, these bacteria could be lost because of the competition with *Nitrospira* sp. and other organotrophic bacteria.

The enrichment of *Nitrospira* sp. and changes over time in other dominants were common features in all non-corroded stainless steels (Figures 9, 10). Although austenitic, martensitic, and duplex stainless steels have different metal compositions and phases, they did not affect the microbial communities in the biofilms formed on these metal surfaces. Environmental factors, such as trace amounts of ions and organic matter, influence microbial communities on non-corroded metal surfaces. On the other hand, the microbial communities in the corrosion products were influenced by metal ions, Fe^2+^ and Cr^2+^, dissolved from the stainless steel, and the iron-oxidizing and toxic-metal-tolerant bacteria survived and were enriched.

## Conclusion

In this study, we demonstrated the MIC of martensitic stainless steel and analyzed microbial community in the corrosion products and the temporal changes in microbial community in the biofilms on the metal surface. Our findings showed that the corrosion of S40300 stainless steel occurred independent of SRB despite the presence of sulfur in the corrosion pits. In many cases, the detection of sulfur in corrosion products is frequently attributed to SRB; however, attention should be paid to the microbial community because sulfur can be present even in the absence of SRB. These findings are useful for the development of appropriate risk assessment methods and diagnosis of MIC.

## Data availability statement

The datasets presented in this study can be found in online repositories. The names of the repository/repositories and accession number(s) can be found at: https://www.ddbj.nig.ac.jp/, DRR381084–DRR381123.

## Author contributions

SW wrote the paper, performed DNA extraction and amplicon sequencing, and analyzed the microbial community data. NE prepared the metal coupons and performed the corrosion behavior analysis and DNA extraction. YM analyzed the metal coupons using a laser microscope. KM, YM, HM, and TS prepared the metal pieces for the immersion study and performed the DNA extraction. All authors contributed to the article and approved the submitted version.

## Funding

This work was supported by Japan Society for the Promotion of Science KAKENHI Grant (grant numbers 17H04719 and 20H02460), Research Grant of ISIJ, MIC Research Grant of NACE-TJS, and a JST PRESTO Grant (grant number JPMJPR21NA).

## Conflict of interest

HM, TS, and NE are employed by INPEX Corporation.

The remaining authors declare that the research was conducted in the absence of any commercial or financial relationships that could be construed as a potential conflict of interest.

## Publisher’s note

All claims expressed in this article are solely those of the authors and do not necessarily represent those of their affiliated organizations, or those of the publisher, the editors and the reviewers. Any product that may be evaluated in this article, or claim that may be made by its manufacturer, is not guaranteed or endorsed by the publisher.
